# Epidemiological Patterns of Animal Bites in the Najafabad, Center of Iran (2012–2017)

**DOI:** 10.5334/aogh.2776

**Published:** 2020-04-07

**Authors:** Sanaz Amiri, Zahra Maleki, Hossein-Ali Nikbakht, Soheil Hassanipour, Hamid Salehiniya, Ali-Reza Ghayour, Hamid Kazemi, Haleh Ghaem

**Affiliations:** 1 Student Research Committee, Shiraz University of Medical Sciences, Shiraz, IR; 2 Social Determinants of Health Research Center, Health Research Institute, Babol University of Medical Sciences, Babol, IR; 3 Gastrointestinal and Liver Diseases Research Center, Guilan University of Medical Sciences, Rasht, IR; 4 Social Determinants of Health Research Center, Birjand University of Medical Sciences, Birjand, IR; 5 Isfahan University of Medical Sciences, Isfahan, IR; 6 Research Center for Health Sciences, Institute of Health, Department of Epidemiology, School of Health, Shiraz University of Medical Sciences, Shiraz, IR

## Abstract

**Background:**

Animal bite due to the risk of rabies is a major public health problem. Rabies is of great importance because of fatalities and economic damage.

**Objective:**

This study was conducted to investigate the epidemiological patterns of animal bite in Najaf Abad during the years of 2012 to 2017.

**Methods:**

This is a cross-sectional study. All records (4,104) were registered in the registration offices of animal bites during the years of 2012 to 2017 at the rabies treatment centres of Najafabad by census method. Demographic characteristics, animal type and sometime clinical patterns of the wounded were examined.

**Results:**

The mean age of the injured was 31.28 ± 15.28 years. Of the 4,104 injured, 3648 (88%) were male and the rest of them were women. In terms of residential area, 3645 people (88%) were in urban areas and the rest were in rural areas in the place of occurrence of bites. Most cases of animal biting occurred in dogs (70.9%) and then cat (24.3%). The most affected part was 51% with shoulder and hand. In this study, the incidence of animal bites is estimated as 100,000 people per year in Najaf Abad in in 2012 it was 206.4, with an increasing trend to 212.9 in 2019 (P < 0.001).

**Conclusion:**

The results of the study showed that most cases of animal bites were related to dogs, urban areas and male sex variables. The incidence of animal bites was also increasing. Due to the importance of this disease and its financial losses, it is recommended that prevention methods should be used to control stray dogs, vaccination of domesticated dogs and to raise awareness of the people.

## Introduction

Animal bites are a significant public health problem, with the majority of bites coming from dogs, cats and humans. These may presented as punctures, abrasions, tears, or avulsions [1]. In general, animals bite occur as a natural, instinctive behavior, especially when they feel threatened or try to get food. An animal bite can cause infection in victims (in both humans and other animals) [2]. One of the most important diseases transmitted by animal bites is rabies.

Rabies is one of the most dangerous viral zoonosis, and all mammals can develop the disease [3,4]. The cause of the disease is a viral nerve-related friend which is belonged to the Rhabdo virus family from the type of Lyssa virus. The disease is mainly transmitted through biting and sometimes through mucous tissue, respiratory tract, placenta, contaminated equipment and transplant organs [5,6]. Rabies is found in two types of epidemiologic condition: of the city type that is principally released by dogs or rarely by cat species, and the wild type whose repository is wolf, fox, raccoon, weasel and bat because of its fatality, an increasing number of animal bites in human, the loss of livestock and economic damage that can produce by rabies, it is very important. About 97% of deaths from rabies are due to dog bite [7]. Rabies is one of the diseases that has been neglected. this disease is preventable by vaccine, and most deaths occur due to the lack of awareness and poor access to health care [8]. Also, animal bites have serious medical consequences such as the risk of rabies, stroke, wound infection and medical expenses for the health system [9,10].

More than 2.5 million people worldwide are exposed to prophylaxis after being exposed to animal bites [11]. In addition to the financial costs of preventing and treating animal cases, the psychological, social consequences of animal bites and the remaining scar can greatly affect the life of an individual and his family [12,13,14]. In Iran, about $12 million annually is spent on rabies vaccine [15]. Due to its fatality, the increasing incidence of animal bites in humans, the loss of livestock and economic damage, rabies is of high importance in the country [16]. In Iran, as in other countries, it is endemic and all provinces of the country are more or less infected with rabies, as the incidence of animal bites has increased in recent years [17,18,19].

To management and evaluate health services, community health assessment is required based on the information about the health problems and disease. So, the lack of epidemiological information is a limiting factor in the prevention and control of the disease. Therefore, it is important to know the most significant factors in the development of this infection, the ways of transmitting the disease, the prevalence of biting and death resulting from it and other factors that need to be taken care of this illness are of particular importance. Also, the wide geographic range, climatic variation and population differences in terms of health and awareness require separate surveys in various parts of the country.

The prediction and implementation of interventions to prevent and reduce animals ‘bite’ and decrease its burden on the health system can help the relevant authorities. Therefore, the aim of this study was to determine the epidemiological patterns of animal bites in Najaf Abad in Isfahan during the years of 2012 to 2017 to help to identify high risk individuals, seasonal and timing patterns of animal bites to prevent the disease.

## Methods

### Setting

In this cross-sectional study, data were collected from 4104 animal bites cases, which were recorded over six years (2012–2017) in health centers of Najafabad in Isfahan province. The data in this study, based on the records from the health center, were obtained from animal bites registration offices. The population of the study consisted of all the cases with animal bites during the mentioned period who were referred to rabies units in Najaf Abad and were under preventive, therapeutic and follow-up measures. Also, patient is a person who has been referred to rabies unit due to animal bites and fear of rabies.

### Data collection

The information that has been collected from an animal’s bite people has included as following: Demographic characteristics (age, sex and occupation), location data of the place of residence (city or village), location of injury (city or village), type of animal, animal status (domestic, wild and feral), the condition of the biting animal after the bite, the characteristics of the damaged site: the affected organ, the extent of the wound, the type of ulcer, bite mode (on clothes or naked body), pattern of injuries (day, month and year), patterns of receiving health services, including vaccination frequency, vaccine Tetanus, rabies, longevity, and animal bites history.

### Statistical analysis

Independent t-test was used to determine the relationship between age and gender, Chi-square test was used for determining the relationship between age and animal bites and ANOVA was used to determine the relationship between age and organs affected. The Cochran Armitage trend test was also used to evaluate the incidence of disease in six years based on the months of the year. For analyzing the data, SPSS software version 18 was used, p-value less than 0.05 was considered significant.

## Results

Of the 4104 injured patients under the study, the mean age of the victims was 31.28 ± 15.34, the age range of the injured varied from 1 to 90 years. There was no significant difference between the mean age of men (31.31 years) and women (31.07) (p = 0.79).

The highest prevalence of animal injuries in both sexes was in the age group of 19–30 years. In this study 3648 (88.9%) were male and the rest were women. Also 3645 (88.8%) cases occurred in the city and 459 (11.2%) took place in the village. The distribution of injuries was also presented in Table 1.

**Table 1:** The distribution of injuries by job category.

Characteristics		Frequency (percent)	Characteristics		Frequency (percent)

Job	Free job	1708 (41.6)	affected organ	Head and Neck	6 (0.1)
	Manual worker	393 (9.6)		Chest and abdomen	11 (0.3)
	Student	348 (9.4)		Shoulder and hand	166 (4)
	Farmer and stockbreeder	369 (9)		Hips and buttocks	19 (0.5)
	employee	271 (6.6)		Thighs and legs	211 (5.1)
	other	979 (23)		no	2997 (73)
animal status	domestic	4081 (99.4)	Vaccination	yes	1841 (44.9)
	Wild and feral	23 (0.6)		no	2263 (55.1)

**Figure 1:**
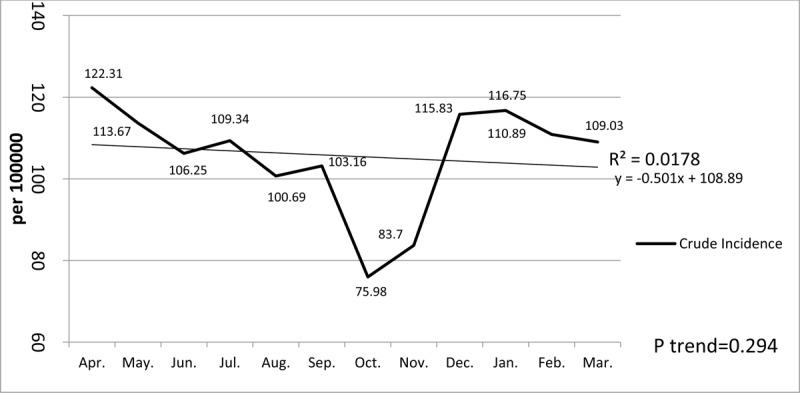
The incidence rate of animal bites, based on months of the year, per hundred thousand people in Najaf Abad (2012–2017).

Regarding bite time, the most cases of animal bite occurred between noon to evening (12–18 hours), in 1492 persons (36.4%), and the lowest cases of injury occurred between night and morning (24–6), in 225 individuals (5.5%). The incidence of animal bites, based on the number of hundreds of thousands of people per month, also showed the lowest incidence was in April with 122.31 and the month of October with 75.98 per hundred thousand people (Figure 1).

**Figure 2:**
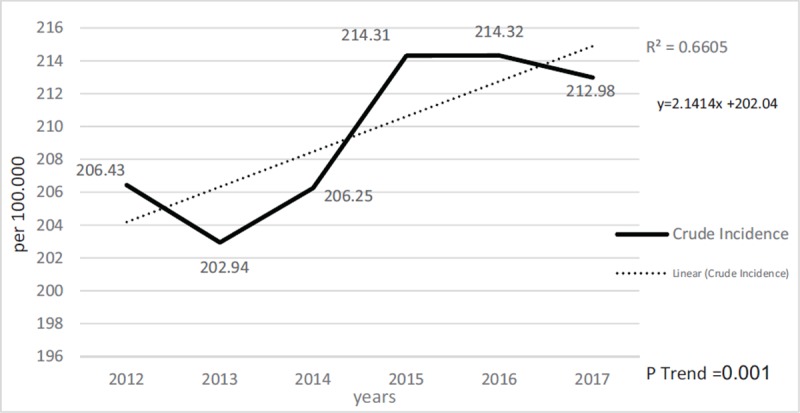
The incidence rate of animal bites per year per hundred thousand people in the Najaf Abad (2012–2017).

**Figure 3:**
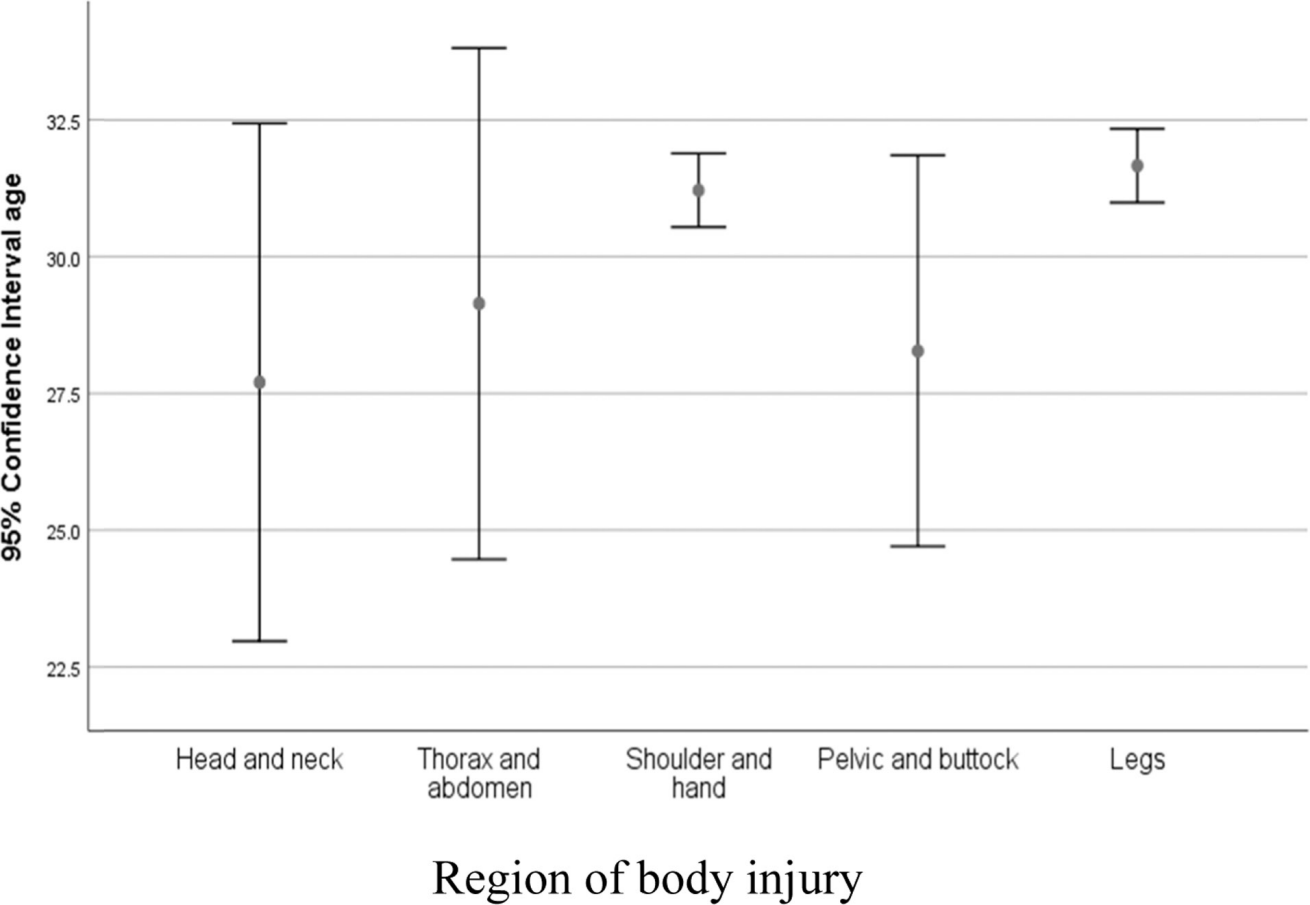
The average age of the victims based on the location of injury from the organs of the injured patients referring to rabies units in the Najaf Abad, Isfahan (2012–2017).

In terms of seasonal distribution, the most cases were in spring with 1108 patients (26%) and the lowest case in the autumn with 892 cases (21%). In this study, the incidence of animal bites in the 100,000 population per year in Najaf Abad in 2012 was 206.4 with an increasing trend of 212.9, in 2017 (Figure 2). The results of Cochran Armitage’s trend test confirmed the elevation in animal bites in Najaf Abad (P < 0.001). The most affected limbs were shoulder and hand, with a frequency of 2107cases (51.3%), followed by thighs and legs with 1810 (44.1%), and pelvis and buttocks, head, neck, chest and abdomen were also in the next rank with 187 (4.5%). There was a significant relationship between age and organ damage (P < 0.001). The lowest average age of the injury in the head and neck was 27.77 ± 17.99 and the highest average age injury in the thigh and legs was 31.66 ± 14.61 (Figure 3). There was a meaningful correlation between age groups and type of animals based on Tukey’s post hoc test (p < 0.001) except for dogs and cats (p = 0.978). Table 2 shows the distribution of the damaging animal species in different age groups in terms of gender, with 1210 (77%) bites in the 19–30-year-old group of dog’s bites, while this value for cats is 72 (40%) cases in the age group under 7 years old. From the point of view of delay in referring to health centers for vaccination, there were 3368 cases (82.1%) without delay, 519 (12%) with one day of delay, and 217 cases (4%) delayed in two or more than two days. Of the bitten subjects (53%), 2197 cases received three times the anti-rabies vaccine and (38%) 1597 received 5 times vaccination. In terms of serum therapy, 3951 (96%) of the injured did not need to receive rabies serum.

**Table 2:** Distribution of injuries by animal species in different age and sex groups (N = 4104).

Age group (year)	Male (N = 3648) N (%)			Female (N = 456) N (%)			P value

	Dog	Cat	Other	Dog	Cat	Other	

**7<**	47 (37)	48 (38)	29 (23)	16 (29)	24 (44)	14 (25)	**0.61**
**7–18**	320 (65)	121 (24)	49 (10)	28 (35)	35 (43)	17 (21)	**<0.001**
**19–30**	1173 (80)	234 (16)	44 (3)	37 (32)	67 (59)	9 (7)	**<0.001**
**31–45**	764 (78)	194 (19)	19 (1)	34 (34)	62 (62)	4 (4)	**<0.001**
**46–60**	332 (75)	104 (23)	6 (1)	29 (38)	44 (57)	3 (3)	**<0.001**
**+61**	112 (68)	48 (29)	4 (2)	17 (51)	16 (48)	0 (0)	**0.07**

## Discussion

The results of this study showed that the incidence of animal bites in the Najaf Abad has been increased; in this survey, the average incidence of animal bites in Najaf Abad per 100,000 people has increased from 204.46 in 2012 to 219.98 in 2017. A Ugandan study also founded an elevating shift to study the long-term trends in the incidence of animal biting and deaths from 2001 to 2015 [20].

A study in Bhutan for the purpose of the occurrence of prophylaxis after exposure from 2005 to 2008 also confirms the increase in the incidence of animal bites [21]. Inside the country, such as the studies that were conducted in 2012 in Ilam and in the study of Nikbakht and colleagues in Babol in 201517,19.,the incidence of animal bites was increasing which is in agreement with this study. In a survey to investigate the epidemiology of animal bites in the Aq Qala in north of Iran between 1998 and 2010, it showed that in the first 5 years of the initiating of the study, animal bites had an increasing and then it had a decreasing trend [22]. Various reasons have been mentioned, such as home improvement, collecting stray dogs, and general education for this decline in that area. Also, studies in the same area as Kolaleh and reported an increasing incidence of animal bites [23]. The reason for this growth can be due to increased quality of data logging, increased awareness of the community about rabies and ways to prevent it, or it may have really increased the incidence of animal bites that need to be explored separately in different studies.

The results of this article also showed that the rate of incidence of animal bites in different seasons is various, and the highest incidence of bites is related to the spring and then the summer season. The results of similar studies confirm this finding [24]. The reasons for this expansion in animal bites could be due to the fact that in the spring, there is more agricultural activity, increased outdoor activities and traveling to rural areas. Of course, the findings of some studies also show photographic results that are not consistent with our study outcomes. These studies indicate that the incidence of animal bites in winter and autumn is higher, which is due to differences in various geographic regions and diversity in the pattern of temperature variation in different area [25].

In this study, men accounted for more than two thirds of the injured, and the male to female ratio was 8 to 1. A study by Riahi et al. in Tabbas showed that 86% of animal bites occurred in men [26]. In the study of Esmailzadeh et al. in 2017 in Fars province, it was observed that 75% of cases of animal bites occurred in men [24]. Similarly, In a study to investigate the epidemiology of animal bites in Ethiopia, it was also manifested that most cases of animal biting occurred in men. (51%) [27]. It seems that the high incidence of injuries in men is due to the more presence of them in the environment for their occupational and non-occupational activities and their exposure and courages. In terms of occupation, free jobs, workers and students had the most cases of animal bites, respectively, which is in agreement with the results of the study of Jafari et al. in Azarshahr [28].

While Charkazi et al. in northern Iran showed the highest percentage of injured students in their study (28.9%). In this investigation, the most frequent animal bites occurred in the age group of 19–30 years [22]. In the study of Majidpour et al. in north of Iran, it was also showed that the majority of cases were between the ages of 19 and 40, which indicates a higher risk for the young population [29]. In Esmailzadeh’s study, the most cases of injury occurred in the age group of 21–30 years (29%), which is consistent with the results of our study [24]. It seems that the high prevalence of animal feeding in adolescents and young people is due to close contact, as well as incitement to animal aggression.

In this survey, the most cases of animal bites occurred by dogs and then in cat. A study in Rafsanjan also depicts similar results [30]. Considering the fact that most biting cases are related to domestic animals, the necessity to train appropriate behavioral skills in dealing with such animals for the high risk populations is obvious. The strain dogs and the vaccination of pets, especially dogs, are also emphasized. Most of the injured limbs were shoulders and arms which were in agreement with the findings of Sheikholeslam et al.’s Study in Rafsanjan, as well as the study of sabouri and his colleagues in Ilam [19,30].

In this study, the most cases of animal bites were reported in urban areas. (88% of cases) while, Nikbakht et al. in their study in Babol founded that most of the animal bites were in rural areas which is in contrast with our study results [17]. Most cases of animal bites in urban areas can be due to better reporting and more accurate animal bites recording. Among the injuries, most of them were superficial and half of the cases of biting were just on the clothes, which is in agreement with the findings of the studies by Nikbakht et al. in Babol [17].

In terms of latency in visiting health centers for post-accident prevention and treatment measures, more than 80% of cases have not been delayed, which indicates good information and high levels of general awareness of rabies and its prevention methods. Of the injured persons, 97% of the subjects did not have a history of vaccination, and 91% of them received anti-rabies vaccine, 45% have recieved tetanus vaccine, as well as 4% of them have received anti-rabies serum injuries. In a study conducted by Jafari et al., in order to investigate the epidemiology of animal bites in the Azarshahr, similar results are founded, of which 3.5% of the injured people received the anti-rabies serum in addition to the vaccine and 4.5% of the injured, they had a history of receiving anti-rabies vaccine [28]. In the study of Charkazi et al. in north of Iran, 72% of the injured person received anti-rabies vaccine were also found to be consistent with our study [22]. This article highlights the importance of informing the general public about rabies.

### The strength and limitation of study

The strength of this study is the existence of complete demographic data of affected people as well as information on the characteristics of the incident and the biting animal, also the end of the biting animal in the long run. From the limitations of this study and other studies it can be ascertained how many percent of the bitten persons are not referred to health centers and their information is not recorded; therefore, such a study is recommended.

## Conclusion

For preventative measures and control for each disease, it is necessary to collect accurate, comprehensive and complete information from epidemiologic patterns of each community. In general, the results of this study, somewhat were in agreement with the findings of some studies in other parts of Iran, but in some cases, there are also discrepancies that need further investigation. The results of this study and other similar surveys showed that the incidence of animal bites in most parts of Iran is increasing. Considering the importance of this disease and its financial and life damages, and since most cases of animal bites have occurred by dogs, it is therefore recommended that prevention methods must be used to control stray dogs, vaccination of domesticated dogs and to raise awareness of the population.
